# Telomere Dysfunction and Aging: Insights From Pulmonary Fibrosis

**DOI:** 10.1093/geroni/igaa057.2701

**Published:** 2020-12-16

**Authors:** Chad Newton

**Affiliations:** University of Texas Southwestern, Dallas, Texas, United States

## Abstract

Telomeres are specialized genomic elements located at the ends of chromosomes that protect protein-encoding DNA from progressive loss during cellular replication. Telomeres shorten with age; therefore, telomere dysfunction is of particular relevance for understanding age-related disease mechanisms. Telomere disorders produce multisystem degenerative organ dysfunction that largely resembles aging phenotypes, including pulmonary fibrosis (PF), emphysema, cirrhosis, bone marry dysfunction, immunosenescence, and premature hair graying. The degree of telomere shortening influences age of disease onset, involved organs, and disease severity. Notably, the most common manifestation of telomere biology disorders is PF. Subsequently, PF is a model for translating telomere biology into clinical practice. I will discuss the genetics of telomere dysfunction and the clinical manifestations that overlap with age-related phenotypes, focusing on PF. Next, I will discuss how short leukocyte telomere length is an informative prognostic, and potentially theragnostic, biomarker in patients with a range of PF subtypes.

